#  Análise espacial da morbimortalidade materna em usuárias do Sistema Único de Saúde no Município do Rio de Janeiro, Brasil, 2014-2016 

**DOI:** 10.1590/0102-311XPT247322

**Published:** 2023-09-18

**Authors:** Heloisa Ferreira dos Santos Correa, Rosa Maria Soares Madeira Domingues, Maria de Fátima Pina

**Affiliations:** 1 Escola Nacional de Saúde Pública Sergio Arouca, Fundação Oswaldo Cruz, Rio de Janeiro, Brasil.; 2 Prefeitura Municipal do Rio de Janeiro, Rio de Janeiro, Brasil.; 3 Instituto Nacional de Infectologia Evandro Chagas, Fundação Oswaldo Cruz, Rio de Janeiro, Brasil.; 4 Instituto de Comunicação e Informação Científica e Tecnológica em Saúde, Fundação Oswaldo Cruz, Rio de Janeiro, Brasil.; 5 Instituto de Investigação e Inovação em Saúde, Universidade do Porto, Porto, Portugal.

**Keywords:** Mortalidade Materna, Morbidade, Sistema de Informação em Saúde, Sistema Único de Saúde, Maternal Mortality, Morbidity, Health Information Systems, Unified Health System, Mortalidad Materna, Morbidad, Sistemas de Información en Salud, Sistema Único de Salud

## Abstract

O objetivo deste estudo é analisar a morbimortalidade materna de mulheres atendidas em hospitais do Sistema Único de Saúde (SUS) no Município do Rio de Janeiro, Brasil, no período de 2014 a 2016. Foi realizado estudo ecológico, por meio da coleta de dados do Sistema de Informações sobre Nascidos Vivos (SINASC), Sistema de Informação sobre Mortalidade (SIM) e Sistema de Informações Hospitalares (SIH/SUS). Para analisar a razão de mortalidade materna (RMM), foram utilizados dados do SIM. Para investigar a morbidade materna, adotaram-se critérios da Organização Mundial da Saúde para estimar as razões de *near miss* materno e de condições potencialmente ameaçadoras à vida. Dados do SINASC foram usados para número de nascidos vivos e caracterização demográfica, social e de acesso a serviço de pré-natal. Para avaliar a associação espacial entre os indicadores RMM, razões de *near miss* materno e condições potencialmente ameaçadoras à vida e os indicadores demográficos, sociais, obstétricos e de acesso obtidos no SINASC, foi calculado o índice de Moran bivariado com nível de 0,05 de significância, por meio do programa GeoDa. No período analisado, a RMM no Município do Rio de Janeiro foi de 94,16/100 mil nascidos vivos, a razão de *near miss* materno de 28,21/1.000 nascidos vivos e a razão de condições potencialmente ameaçadoras à vida de 34,31/1.000 nascidos vivos. Casos de condições potencialmente ameaçadoras à vida foram utilizados pela primeira vez neste estudo e apresentaram diagnósticos de internação e procedimentos realizados mais condizentes com o perfil de mortalidade materna no Município do Rio de Janeiro. Houve associação significativa entre RMM e percentual de nascidos vivos no SUS, razão de condições potencialmente ameaçadoras à vida e percentual de nascidos vivos no SUS e razão de condições potencialmente ameaçadoras à vida e ser solteira.

## Abstract (en)

The objective of this study is to analyze the maternal morbidity and mortality of women treated in hospitals of the Brazilian Unified National Health System (SUS) in the city of Rio de Janeiro in the period 2014-2016. An ecological study was conducted using data from the Brazilian Information System on Live Birth (SINASC), the Brazilian Mortality Information System (SIM), and the Brazilian Hospital Information System (SIH/SUS). For the analysis of the maternal mortality ratio (MMR), data from the SIM were used. For the analysis of maternal morbidity, World Health Organization criteria were used to estimate the ratios of maternal near miss and potentially life-threatening conditions. SINASC was used to retrieve data on the number of live births, for demographic characterization, social aspects, and access to prenatal care. To evaluate the spatial association between the indicators MMR, ratios of maternal near miss, and potentially life-threatening conditions and the demographic, social, obstetric, and access indicators, obtained from SINASC, the bivariate Moran Index was estimated with a significance level of 0.05, using the GeoDa program. In the period analyzed, the MMR in the Rio de Janeiro was 94.16/100,000 live births, the ratio of maternal near miss was 28.21/1,000 live births, and the potentially life-threatening conditions was 34.31/1,000 live births. Cases of potentially life-threatening conditions were used for the first time in this study and presented diagnoses and procedures during hospitalization more consistent with the maternal mortality profile in the city of Rio de Janeiro. There was a significant association between MMR and percentage of live births in SUS, potentially life-threatening conditions and percentage of live births in SUS, and potentially life-threatening conditions and being single.

## Abstract (es)

El objetivo de este estudio es analizar la morbimortalidad materna de las mujeres atendidas en hospitales del Sistema Único de Salud (SUS) del municipio de Rio de Janeiro, Brasil, en el período 2014-2016. Fue realizado un estudio ecológico, por medio del uso de datos del Sistema de Información sobre Nacidos Vivos (SINASC), Sistema de Información sobre Mortalidad (SIM) y del Sistema de Información Hospitalaria (SIH/SUS). Para el análisis de la razón de mortalidad materna (RMM) se utilizaron los datos SIM. Para el análisis de la morbilidad materna se utilizaron los criterios de la Organización Mundial de la Salud para estimar las razón de *near miss* materno y de condiciones potencialmente amenazantes a la vida. Para el número de nacidos vivos y la información demográfica, social y de acceso al servicio de atención prenatal fueron utilizados datos del SINASC. Para evaluar la asociación espacial entre los indicadores RMM, razon de *near miss* materno y razon de condiciones potencialmente amenazantes a la vida y los indicadores demográficos, sociales, obstétricos y de accesos obtenidos en el SINASC fue calculado el Índice de Moran bivariado con nivel de 0,05 de significación, usando el programa GeoDa. En el período analizado, la RMM en el municipio de Rio de Janeiro fue de 94,16/100.000 nascidos vivos, la razón de *near miss* materno de 28,21/1.000 nascidos vivos y la razón de condiciones potencialmente amenazantes a la vida de 34,31/1.000 nascidos vivos. Los casos de condiciones potencialmente amenazantes a la vida se utilizaron por primera vez en este estudio y presentaron diagnósticos de hospitalización y procedimientos realizados más acordes con el perfil de mortalidad materna en el Municipio de Rio de Janeiro Hubo una asociación significativa entre RMM y el porcentaje de nacidos vivos en el SUS, razón de condiciones potencialmente amenazantes a la vida y el porcentaje de nacidos vivos en el SUS y razón de condiciones potencialmente amenazantes a la vida y ser soltera.

## Introdução

A mortalidade materna - morte de uma mulher durante a gestação ou até 42 dias após seu término, devido a qualquer circunstância relacionada ou agravada pela gravidez, exceto as causas acidentais ou incidentais ^1^ - permanece como um dos piores indicadores de saúde em locais com recursos limitados, sendo a grande maioria dos óbitos potencialmente evitável pela atuação oportuna dos serviços e do sistema de saúde.

Entretanto, mortes maternas são raras, em número absoluto, o que dificulta sua análise estatística, especialmente no âmbito local dos serviços ^2^. Por essa razão, o estudo da morbidade materna tem sido recomendado como estratégia complementar importante para avaliar e melhorar a qualidade da assistência à mulher no período gravídico-puerperal ^3^^,^^4^^,^^5^, já que casos de morbidade grave são mais frequentes e compartilham dos mesmos determinantes do óbito materno.

No espectro da morbidade materna, as condições potencialmente ameaçadoras à vida e os casos de *near miss* materno (mulher que vivencia situações ameaçadoras à vida durante a gestação, o parto ou até 42 dias após o término da gravidez e sobrevive) situam-se no extremo da gravidade e representam circunstâncias que antecedem o óbito materno. A Organização Mundial da Saúde (OMS) recomenda a utilização de critérios padronizados para a classificação desses eventos - condições potencialmente ameaçadoras à vida e *near miss* materno -, visando comparar dados entre países e serviços ou monitorar tais informações no mesmo serviço ou país ao longo do tempo ^1^. A padronização dos critérios viabilizou a comparação entre diversas regiões, pois anteriormente eram utilizados diferentes critérios elaborados por distintos pesquisadores ^6^.

O Sistema de Informações Hospitalares do Sistema Único de Saúde (SIH/SUS) é o único meio disponível que contém informações sobre morbidade materna no setor público. Entretanto, pesquisas nacionais evidenciam incerteza quanto à sua utilização para o estudo da morbidade materna. Enquanto algumas indicam as vantagens do SIH, como a elevada cobertura de parto hospitalar no país e a disponibilidade de informações, outras apontam sua utilização prioritária para o pagamento das internações, com dúvidas sobre a qualidade da informação, além da dificuldade de operacionalização dos critérios recomendados pela OMS com os dados disponíveis no SIH/SUS ^7^^,^^8^^,^^9^.

O Município do Rio de Janeiro apresenta uma razão de mortalidade materna elevada e praticamente estável, variando de 72,64 a 82,82 por 100 mil nascidos vivos entre os anos 2009 e 2017, apesar da cobertura de assistência pré-natal e parto praticamente universal, indicando existência de problemas na qualidade dos serviços prestados ^10^.

Considerando que o espaço geográfico é uma dimensão de análise fundamental para a saúde, já que todos os acontecimentos - nascimentos, óbitos, internações hospitalares, adoecimento etc. - ocorrem em uma localização e podem ser georreferenciados ^11^, o padrão de distribuição geográfica da morbimortalidade e suas relações com fatores socioambientais podem ser analisados para a detecção de aglomerados espaciais ou espaçotemporais, o monitoramento ambiental, o planejamento e a avaliação, bem como para apoiar a tomada de decisões quando há identificação de tendências ^12^. O objetivo da análise espacial é incorporar o espaço à análise, baseando-se em um dos conceitos básicos da análise espacial, a primeira lei da geografia, que é definida como: “*todas as coisas são parecidas, mas coisas mais próximas se parecem mais que coisas mais distantes*” ^13^ (p. 236).

No Município do Rio de Janeiro, os bairros são agregados em 33 regiões administrativas (RA), que, por sua vez, são agrupadas em dez áreas de planejamento da saúde (AP). Essa divisão territorial tem por finalidade a gestão de recursos de acordo com as realidades de cada AP, definindo as necessidades prioritárias de cada grupo populacional específico. Entretanto, em uma mesma AP, as RA têm características sociodemográficas distintas, o que demanda a observação constante das necessidades de saúde da população e a alocação de recursos humanos e financeiros.

Dessa forma, considerando a relevância do estudo da morbimortalidade materna para planejamento e avaliação dos serviços de cuidado obstétricos, este artigo tem por objetivo analisar a morbimortalidade materna de mulheres atendidas no SUS no Município do Rio de Janeiro no período de 2014 a 2016 por meio da análise espacial.

## Métodos

Este é um estudo ecológico, que tem como unidades de análise as 33 RA do Município do Rio de Janeiro. Foram utilizados três sistemas de informação - Sistema de Informações de Nascidos Vivos (SINASC), Sistema de Informação sobre Mortalidade (SIM) e Sistema de Informações Hospitalares (SIH/SUS) - para a identificação, respectivamente, de nascidos vivos, óbitos maternos e casos de condições potencialmente ameaçadoras à vida e *near miss* maternal ocorridos em maternidades públicas do Município do Rio de Janeiro. Como casos de óbito materno são eventos pouco frequentes, foram coletados dados dos três anos mais recentes com informações disponíveis (2014-2016) na época de realização do estudo, visando atenuar a instabilidade estatística nas taxas, resultante do problema dos pequenos números.

Foram excluídos os nascimentos e óbitos ocorridos em maternidades privadas, porque o SIH é utilizado apenas em maternidades públicas e a manutenção de casos que aconteceram em maternidades privadas resultaria em estimativas enviesadas, visto que casos de *near miss* materno e condições potencialmente ameaçadoras à vida ocorridos em maternidades privadas não seriam incluídos no numerador dos indicadores calculados. O Município do Rio de Janeiro não dispõe de maternidades conveniadas ao SUS, portanto, todos os partos realizados em maternidades privadas não recebem financiamento público.

O número de nascidos vivos no período de 2014 a 2016 foi obtido da base nominal do SINASC, bem como características dos nascidos vivos nesse período, e utilizado no cálculo, por região administrativa, dos seguintes indicadores que refletem situações de maior vulnerabilidade ao óbito materno: (i) demográficos: proporção de mães de nascidos vivos com idade de 10 a 19 anos (mães adolescentes), proporção de nascidos vivos de raça/cor preta ou parda, proporção de nascidos vivos de mães com estado civil solteira; (ii) sociais: proporção de mães de nascidos vivos com até nove anos de estudo, correspondendo ao Ensino Fundamental I e II; (iii) obstétricos: proporção de nascidos vivos de mães primíparas, proporção de nascidos vivos por cesariana; (iv) acesso a serviços de saúde: proporção de mães de nascidos vivos que receberam assistência pré-natal; proporção de mães de nascidos vivos com início do pré-natal tardio, após o primeiro trimestre gestacional.

Para identificar casos de óbito materno de mulheres internadas no SUS, foi utilizado o SIM. Selecionaram-se, para o período de 2014 a 2016, todos os casos de óbito materno de residentes no Município do Rio de Janeiro cujo parto tenha ocorrido em maternidade pública. Para isso, relacionou-se a base do SIM com a do SINASC por meio da busca manual dos casos de óbito materno, com exclusão de todas as mulheres que tiveram filhos em maternidades privadas. Para identificar essas mulheres no SINASC, foram utilizados o nome e a data de nascimento da mulher. Óbitos ocorridos em serviços públicos do Município de Rio de Janeiro e que não foram localizados no SINASC, provavelmente correspondendo a óbitos ocorridos na gestação ou em gestações que tiveram como desfecho um óbito fetal, foram considerados como casos de óbitos de usuárias do SUS.

Para avaliar a cobertura do SIH/SUS para partos, foi calculada a proporção de partos de nascidos vivos registrados no SIH/SUS segundo RA de residência da mulher em relação ao total de nascidos vivos em maternidades públicas, por RA, registrado no SINASC. Cálculo semelhante foi realizado para a cobertura de óbitos fetais, calculando-se a proporção de partos com desfecho óbito fetal registrados no SIH/SUS em relação ao total de óbitos fetais ocorridos em maternidades públicas, por RA, registrados no SIM.

Para identificar casos de *near miss* materno e de condições potencialmente ameaçadoras à vida, utilizou-se o SIH/SUS relativo ao período de 2014 a 2016. Para selecionar os diagnósticos e procedimentos para identificação de casos de *near miss* materno, foram adotados os critérios propostos por Nakamura-Pereira et al. ^7^, que buscaram operacionalizar a definição de *near miss* materno da OMS por meio dos dados disponíveis no SIH/SUS. Procedimentos utilizados pelos autores, que não constavam da tabela de procedimentos do SIH/SUS em 2014-2016, foram excluídos. No Quadro 1, são apresentados os diagnósticos e procedimentos utilizados para identificar casos de *near miss* materno no SIH/SUS neste estudo.

**Quadro 1:** Diagnósticos e procedimentos do Sistema de Informação Hospitalar do Sistema Único de Saúde (SIH/SUS) utilizados para a identificação de casos de *near miss* materno e de condições potencialmente ameaçadoras à vida materna segundo critérios da Organização Mundial da Saúde.

CRITÉRIO	DIAGNÓSTICOS, CONFORME A CID-10	PROCEDIMENTOS (SUS)
*NEAR MISS* MATERNO *		
Disfunção cardiovascular	• Choque hipovolêmico; depleção de volume [D62; E86; O75.1; R57.1.; O03.1; O03.6; O04.1; O04.6; O05.1; O05.6; O06.1; O06.6; O07.1; O07.6; O08.1; 044.1; O45; O45.8; O45.9; O46; O46.8; O46.9; O67; O67.8; O67.9; O69.4; O71.0; O71.1; O72; O72.0; O72.1; O72.2; O90.0] • Outras formas de choque [R57; R57.8; R57.9; T79.4; T81.1; T88.2; T88.6] • Infecção; sepse; aborto complicado por infecção do trato genital; peritonite; salpingite [A02.1; A22.7; A26.7; A32.7; A40; A40.0; A40.1; A40.2; A40.3; A40.8; A40.9; A41; A41.0; A41.1; A41.2; A41.3; A41.4; A41.5; A41.8; A41.9; A42.7; A54.8; B37.7; K35.0; K35.9; K65.0; K65.8; K65.9; M86.9; N70.0; N70.9; N71.0; N73.3; N73.5; O03.0; O03.5; O04.0; O04.5; O05.0; O05.5; O06.0; O06.5; O07.0; O07.5; O08.0; O08.2; O08.3; O41.1; O75.3; O85; O86; O86.0; O86.8; O88.3; R10; T80.2] • Insuficiência cardíaca [I11.0; I13.0; I13.2; I50; I50.0; I50.1; I50.9; R57.0] • Cardiomiopatia [I42.0; I42.1; I42.8; I42.9; I43.8; O90.3] • Parada cardíaca [I46; I46.0; I46.9; O75.4] • Complicações cardiovasculares da anestesia durante gravidez, parto e puerpério [O29; O29.0; O29.1; O29.3; O29.5; O29.8; O29.9; O74; O74.2; O74.4; O74.6; O74.8; O74.9; O89; O89.1; O89.3; O89.5; O89.8; O89.9; T88.3; T88.5] • Tireotoxicose; distúrbio metabólico consequente a aborto e à gravidez ectópica e molar [E05; E05.0; E05.1; E05.2; E05.3; E05.4; E05.5; E05.8; E05.9; E06.0; E06.2; O08.5]	• Tratamento de insuficiência cardíaca [0303060212] • Tratamento de crise hipertensiva [0303060107] • Tratamento de arritmias [03030600026] • Tratamento do choque cardiogênico [0303060069] • Tratamento de hipertensão secundária [0303060182] • Tratamento de parada cardíaca com ressuscitação bem-sucedida [0303060255] • Tratamento de choque hipovolêmico [0303060077] • Tratamento de complicações relacionadas predominantemente ao puerpério [0303100010] • Tratamento de outras doenças bacterianas [0303010037] • Tratamento de doenças inflamatórias dos órgãos pélvicos femininos [0303150033] • Tratamento cirúrgico de peritonite [0407040250] • Tratamento de doenças do peritônio [0303070080] • Histerorrafia [0409060160] • Tratamento cirúrgico da inversão uterina aguda [0411010085] • Tratamento com cirurgias múltiplas [045010012] • Drenagem de hematoma/abscesso pré-peritoneal [0407040030] • Drenagem de abscesso pélvico [0407040013] • Drenagem de abscesso [0401010031] • Incisão e drenagem de abscesso [0401010104] • Tratamento do choque anafilático [0303060050]
Disfunção respiratória	• Edema pulmonar [J81] • Embolia pulmonar; aborto complicado por embolia [I26; I26.0; I26.9; O03.2; O03.7; O04.2; O04.7; O05.2; O05.7; O06.2; O06.7; O07.2; O07.7; O08.2; O88; O88.0; O88.1; O88.2; O88.3] • Complicações pulmonares da anestesia durante gravidez, parto e puerpério [O29; O29.3; O29.5; O29.6; O29.8; O29.9; O74; O74.0; O74.1; O74.7; O89; O89.0; O89.6; T88.4; T88.5] • Insuficiência respiratória [J80; J96; J96.0; J96.9; R09.2]	• Tratamento de edema agudo de pulmão [0303060131] • Tratamento de embolia pulmonar [0303060140] • Tratamento de outras doenças do aparelho respiratório [0303140135]
Disfunção renal	• Anúria e oligúria [R34] • Insuficiência renal pós-parto e após aborto e gravidez ectópica e molar [O08.4; O90.4] • Insuficiência renal aguda [E72.2; I12.0; I13.1; I13.2; N17; N17.0; N17.1; N17.2; N17.8; N17.9; N18.0]	• Hemodiálise [03050100420305010093, 0305010107, 0305010115, 0305010123, 0305010131] ** • Tratamento de insuficiência renal aguda [0305020048]
Disfunção hematológica ou hemostática	• Coagulação intravascular disseminada; defeito de coagulação [D65; D68; D68.9; D69.4; D69.5; D69.6; D82.0; O45.0; O46.0; O67.0; O72.3]	• Tratamento de defeitos da coagulação [0303020067] • Transfusão de concentrados de plaquetas [0306020076] • Transfusão de crioprecipitado [0306020084] • Transfusão de plaquetas por aférese [0306020092] • Transfusão de plasma fresco [0306020106] • Transfusão de plasma isento de crioprecipitado [0306020114]
Disfunção hepática	• Insuficiência hepática [K72; K72.0; K72.9] • Transtornos do fígado e hepatites virais complicando a gravidez [O26.6; O98.4] • Icterícia não especificada [R17]	• Tratamento de doenças do fígado [0303070072] • Tratamento de hepatites virais [0303010118]
Disfunção neurológica	• Sonolência, estupor e coma [R40] • Coma não especificado [R402] • Coma hipoglicêmico, não diabético [E15] • Hemorragia intracerebral; acidente cerebrovascular; trombose venosa cerebral na gravidez [G93.6; I60; I60.0; I60.1; I60.2; I60.3; I60.4; I60.5; I60.6; I60.7; I60.9; I61; I61.0; I61.1; I61.2; I61.3; I61.4; I61.5; I61.6; I61.8; I61.9; I64; I69.1; O22.5] • Eclâmpsia [O15, O15.0, O15.1, O15.2, O15.9] • Epilepsia [G40.0, G40.1, G40.2, G40.3, G40.4, G40.5, G40.6, G40.7, G40.8, G40.9] • Complicações do sistema nervoso central relacionadas à anestesia na gravidez, parto e puerpério [O29.2; O74.3; O89.2] • Diabetes mellitus com coma ou cetoacidose [E10.0; E10.1; E11.0; E11.1; E12.0; E12.1; E13.0; E13.1; E14.0; E14.1]	• Diária em unidade de terapia intensiva adulto [0802010083, 0802010091, 0802010105] • Tratamento conservador da hemorragia cerebral [0303040076] • Tratamento da eclâmpsia [0303100028] • Tratamento de crises epilépticas não controladas [0303040165]
Outros		• Histerectomia total, subtotal, puerperal ou com anexectomia [0409060135, 0409060127, 0411020030, 0409060119, respectivamente]
CONDIÇÕES POTENCIALMENTE AMEAÇADORAS À VIDA MATERNA **		
Distúrbios hemorrágicos		
Descolamento prematuro de placenta Placenta acreta, increta ou percreta Prenhez ectópica Hemorragia pós-parto Rotura uterina	• Descolamento prematuro de placenta [O450; O458; O459] • Placenta acreta, increta ou percreta [O720; O730] • Prenhez ectópica [O008; O009; O081] • Hemorragia pós-parto [O720; O721; O722; O723] • Rotura uterina [O710; O711]	• Tratamento cirúrgico de gravidez ectópica [411020048]
Distúrbios hipertensivos		
Pré-eclâmpsia grave Eclâmpsia Hipertensão grave Encefalopatia hipertensiva Síndrome HELLP	• Pré-eclâmpsia grave [O11; O141] • Eclâmpsia [O150; O151; O152; O159] • Encefalopatia hipertensiva [I674] • Síndrome HELLP [O141]	• Tratamento de edema, proteinúria e transtornos hipertensivos na gravidez parto e puerpério [303100036] • Tratamento de eclâmpsia [303100028]
Outros distúrbios sistêmicos		
Endometrite Edema pulmonar Insuficiência respiratória Convulsões Sepse Choque Trombocitopenia < 100.000 Crise tireotóxica	• Endometrite [O85] • Edema pulmonar [J81] • Insuficiência respiratória [J960; J969] • Convulsões [R568] • Sepse [A418; A419; O085; O083; O751] • Choque [R570; R571; R578; R579; T811] • Trombocitopenia < 100.000 [D695; D696] • Crise tireotóxica [E054; E055; E058, E059]	• Tratamento de edema agudo de pulmão [303060131] • Tratamento de choque cardiogênico [303060069] • Tratamento de choque hipovolêmico [303060077] • Tratamento de defeitos da coagulação, púrpura e outras afecções hemorrágicas [303020067]
Indicadores de gravidade de manejo		
Transfusão sanguínea	• Transfusão sanguínea [Z513]	• Transfusão de concentrado de hemácias [306020068] • Transfusão de concentrado de plaquetas [306020076] • Transfusão de crioprecipitado [306020084] • Transfusão de plaquetas por aférese [306020092] • Transfusão de plasma fresco [306020106] • Transfusão de plasma isento de crioprecipitado [306020114] • Transfusão de sangue/componentes irradiados [306020122] • Transfusão de substituição/troca [306020130] • Transfusão de unidade de sangue total [306020149]
Acesso venoso central		
Histerectomia	• Histerectomia [O822]	• Histerectomia, total, subtotal, puerperal, com ou sem anexectomia [411020030; 409060100; 409060127; 409060135; 416060110]
Admissão à unidade de tratamento intensivo		• Diária de unidade de terapia intensiva adulto [802010083; 802010091; 802010105]
Internação hospitalar prolongada (> 7 dias pós-parto)		
Intubação não anestésica	• Intubação não anestésica [O296; O747; O896]	
Retorno à sala operatória (centro cirúrgico)	• Retorno à sala operatória (centro cirúrgico) [Z489]	
Intervenção cirúrgica		• Tratamento c/ cirurgias múltiplas [415010012] • Laparotomia exploradora [407040161]

CID-10: *Classificação Estatística Internacional de Doenças e Problemas Relacionados com a Saúde*, 10ª revisão.

* Nakamura-Pereira et al. ^7^, com atualização dos procedimentos vigentes no período 2014-2016;

** Foram retirados os procedimentos 0305010050, 0305010069, 0305010077 e 0305010085, que correspondiam à hemodiálise, mas não estavam mais vigentes em 2014-2016;

*** Elaboração própria.

Não foram encontrados estudos nacionais que tenham utilizado o SIH/SUS para encontrar casos de condições potencialmente ameaçadoras à vida. Assim, como base, foram usados os diagnósticos e procedimentos empregados por Nakamura-Pereira et al. ^7^ para selecionar os casos de *near miss* materno, com exclusão e acréscimo de diagnósticos e procedimentos visando atender aos critérios de definição de caso de condições potencialmente ameaçadoras à vida da OMS. Essa seleção foi feita a partir da identificação inicial de códigos da *Classificação Estatística Internacional de Doenças e Problemas Relacionados com a Saúde*, 10ª revisão (CID-10), e de procedimentos realizados pela autora, seguida de consulta a um médico obstetra e a um médico epidemiologista. Os códigos e procedimentos utilizados na seleção dos casos de condições potencialmente ameaçadoras à vida estão no Quadro 1.

Para identificar casos de *near miss* materno e condições potencialmente ameaçadoras à vida no SIH/SUS, foram seguidas as seguintes etapas:

(1) Seleção das internações de mulheres em idade fértil (10 a 49 anos), residentes do Município do Rio de Janeiro, cujas datas de admissão hospitalar tenham ocorrido entre 1º de janeiro de 2014 e 31 de dezembro de 2016;

(2) Exclusão das solicitações de cobrança (Autorização de Internação Hospitalar - AIH) rejeitadas;

(3) Seleção das internações com especialidade obstetrícia ou com diagnóstico principal e/ou secundário do capítulo XV - Gravidez, parto e puerpério - da CID-10;

(4) Identificação de internações com campos “diagnóstico principal”, diagnóstico secundário” ou “procedimento realizado” com codificações compatíveis com condições potencialmente ameaçadoras à vida e *near miss* materno descritos no Quadro 1. Foram consideradas casos de *near miss* materno e de condições potencialmente ameaçadoras à vida internações que atendessem a pelo menos um critério diagnóstico de *near miss* materno ou condições potencialmente ameaçadoras à vida apresentado no Quadro 1;

(5) Identificação de internações em unidade de terapia intensiva (UTI) por meio da variável “enfermaria” do banco de dados do SIH/SUS para identificação de casos de *near miss* materno e condições potencialmente ameaçadoras à vida; e

(6) Exclusão de duplicidades.

A partir dos casos identificados de *near miss* materno, de condições potencialmente ameaçadoras à vida e de óbitos maternos ocorridos em maternidades públicas, foram estimados os seguintes indicadores por RA do Município do Rio de Janeiro:

(a) Razão de mortalidade materna (RMM): casos de óbito materno/100 mil nascidos vivos;

(b) Razão de *near miss* materno: casos de *near miss* materno/1.000 nascidos vivos; e

(c) Razão de condições potencialmente ameaçadoras à vida: casos de condições potencialmente ameaçadoras à vida/1.000 nascidos vivos.

Na última etapa, foi verificada a existência de associação espacial dos indicadores propostos pela OMS com as características demográficas, sociais, obstétricas e de acesso a serviços das mulheres no Município do Rio de Janeiro, utilizando a RA como unidade territorial. Na fase inicial de análise exploratória, foram feitas a visualização dos dados sob a forma de gráficos e mapas e a identificação de padrões de dependência espacial ^14^^,^^15^ por meio do software de uso livre TerraView, versão 4.2.2 (http://www.dpi.inpe.br/terraview). Posteriormente, avaliou-se a relação entre os indicadores RMM, razão de *near miss* materno e razão de condições potencialmente ameaçadoras à vida e os indicadores demográficos, sociais, obstétricos e de acesso obtidos no SINASC mediante a correlação espacial bivariada (índice de Moran bivariado) com nível de 0,05 de significância, por meio do programa GeoDa (https://spatial.uchicago.edu/software). O índice de Moran I varia de -1 a +1 e mede a existência de dependência espacial na distribuição dos dados ^14^, ou seja, verifica se os eventos estão distribuídos aleatoriamente no espaço ou se um fenômeno que ocorre em um lugar está associado a episódios que acontecem na vizinhança desse local. Valores próximos de zero indicam aleatoriedade espacial; valores positivos indicam autocorrelação espacial positiva; e valores negativos indicam autocorrelação espacial negativa.

Este projeto foi aprovado pelo Comitê de Ética em Pesquisa da Escola Nacional de Saúde Pública Sergio Arouca/Fundação Oswaldo Cruz (ENSP/Fiocruz, parecer nº 2.620.129) e da Secretaria Municipal da Saúde do Rio de Janeiro (SMS-RJ, parecer nº 2.792.396), pois utilizou base de dados identificados. Não foi necessário assinar Termo de Consentimento Livre e Esclarecido por se tratar de análise de dados secundários. Todos os cuidados foram adotados visando garantir o sigilo e a confidencialidade das informações processadas.

## Resultados

Dos 263.511 partos de nascidos vivos no Município do Rio de Janeiro no período de 2014 a 2016, 160.361 (60,86%) foram de mulheres usuárias do SUS, com variação de 25% a 91%, sendo os maiores valores observados nas RA Rocinha, Jacarezinho, Complexo do Alemão e Complexo da Maré, onde se concentram populações de baixa renda.

Dos nascidos em maternidades SUS, 75,82% das mães se autodeclararam pardas ou pretas, 82,55% estavam solteiras e 36,87% tinham até nove anos de estudo. Quase 40% estavam na primeira gestação e 22,76% eram mães adolescentes. Mais de 90% tiveram assistência pré-natal, mas 23,54% iniciaram os cuidados tardiamente, no segundo ou terceiro trimestre gestacional. Mais de um terço (36,02%) dessas mulheres apresentaram cesariana como desfecho da gestação atual.

Dos 7.367 óbitos de mulheres em idade fértil no período de 2014 a 2016, foram identificados 191 óbitos maternos de residentes do Município do Rio de Janeiro. Após exclusão de oito óbitos ocorridos em outros municípios, nove ocorridos em domicílio ou via pública/outros e 23 em hospitais particulares e militares do Município do Rio de Janeiro, foram incluídos nesta análise 151 óbitos de residentes do Município do Rio de Janeiro que aconteceram em unidades de saúde SUS no período de 2014 a 2016.

Foram identificadas 121.578 internações para parto de mulheres residentes no Município do Rio de Janeiro no SIH/SUS. A média de cobertura do SIH/SUS para partos de nascidos vivos de residentes no Município do Rio de Janeiro identificados no SINASC foi de 75,82%, variando de 0,63% no Complexo do Alemão a 123,69% na Lagoa. Já a média de cobertura do SIH/SUS para partos de óbitos fetais de residentes no Município do Rio de Janeiro identificados no SIM foi de 68,68%, variando de 0% na RA Complexo do Alemão a 197,56% na RA Ramos.

Das 408.869 internações de mulheres em idade fértil ocorridas no período de 2014 a 2016 registradas no SIH/SUS, foram identificados 4.524 casos de *near miss* materno e 5.502 casos de condições potencialmente ameaçadores à vida em mulheres residentes no Município do Rio de Janeiro. Dos 4.524 casos de *near miss* materno, 3.492 foram selecionados pelo critério diagnóstico, 2.423 pelo critério procedimento realizado e 28 pela internação em UTI. Já para os casos de condições potencialmente ameaçadores à vida, foram selecionados 3.928 casos pelo critério diagnóstico, 2.270 pelo procedimento realizado e 28 pela internação em UTI.

Entre os critérios diagnósticos, o grupo das causas infecciosas (33,62%) e o dos transtornos hemorrágicos (31,74%) foram os mais frequentes para os casos de *near miss* materno, enquanto para condições potencialmente ameaçadores à vida os grupos mais frequentes foram transtornos hipertensivos (40,35%), transtornos hemorrágicos (21,77%), infecções (13,67%) e demais causas (24,21%). Quanto aos procedimentos, o mais frequente na seleção dos casos de *near miss* materno foi “tratamento de complicações relacionadas predominantemente ao puerpério” (78,25%), seguido pela “histerorrafia” (13,21%). Para os casos de condições potencialmente ameaçadores à vida, os procedimentos “tratamento de edema, proteinúria e transtornos hipertensivos na gravidez parto e puerpério” (44,85%) e “tratamento cirúrgico de gravidez ectópica” (33,22%) foram os mais frequentes.

**Figura 1:**
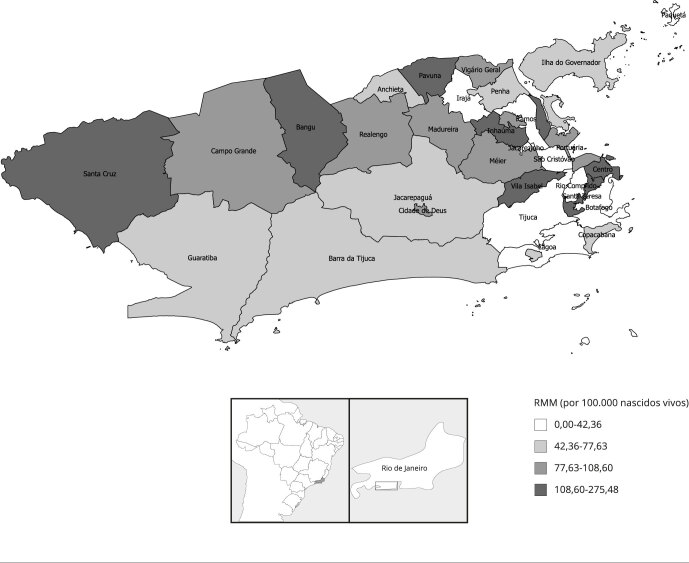
Razão de mortalidade materna (RMM) por região administrativa do Município do Rio de Janeiro, Brasil, 2014-2016.

**Figura 2:**
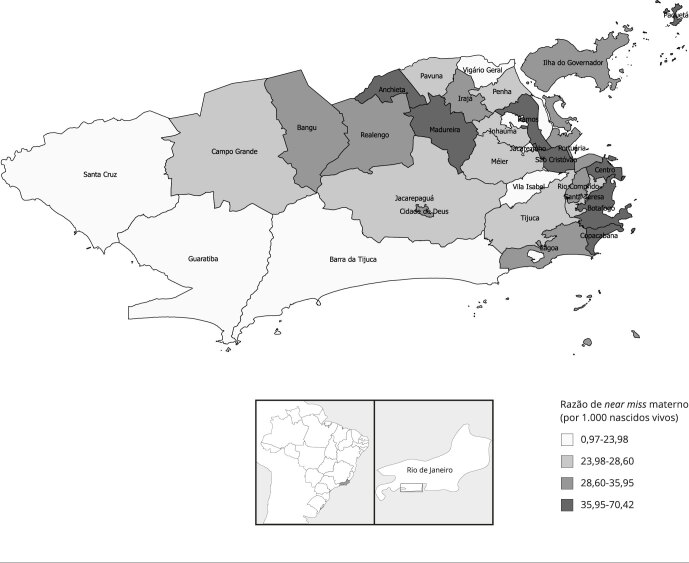
Razão de *near miss* materno por região administrativa do Município do Rio de Janeiro, Brasil, 2014-2016.

No período estudado, a RMM no Município do Rio de Janeiro foi de 94,16/100 mil nascidos vivos, enquanto a razão de *near miss* materno foi de 28,21/1.000 nascidos vivos e a razão de condições potencialmente ameaçadores à vida de 34,31/1.000 nascidos vivos para o Município do Rio de Janeiro. As Figuras 1, 2 e 3 apresentam os indicadores de morbimortalidade materna por RA do Município do Rio de Janeiro. Os valores mais elevados de RMM foram observados nas RA Centro e Santa Teresa; enquanto os maiores valores de razão de *near miss* materno, nas RA Paquetá e Ramos. Já os valores mais altos de razão condições potencialmente ameaçadores à vida foram encontrados nas RA Cidade de Deus e Barra da Tijuca.

**Figura 3:**
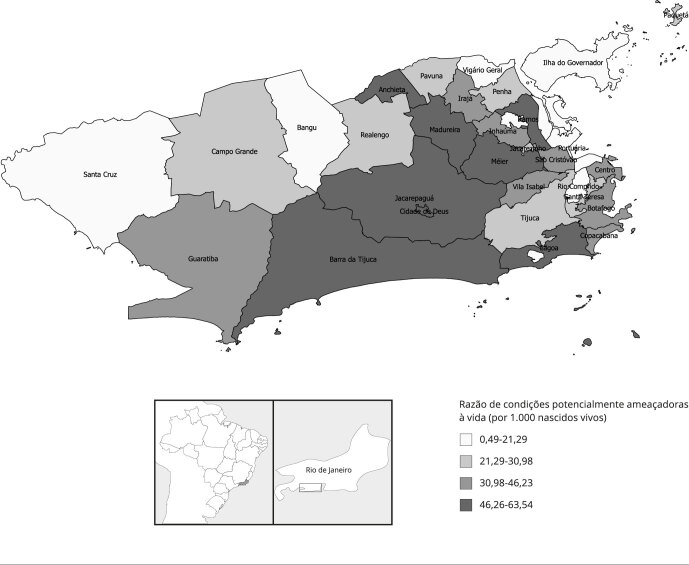
Razão de condições potencialmente ameaçadoras à vida materna por região administrativa do Município do Rio de Janeiro, Brasil, 2014-2016.

A Tabela 1 mostra o índice de Moran bivariado calculado para a relação entre RMM, razão de *near miss* materno e razão de condições potencialmente ameaçadores à vida por RA e os indicadores demográficos, sociais, obstétricos e de acesso ao pré-natal. Índices entre 0 e 1 indicam que existe uma relação direta entre as razões e os indicadores. O percentual de nascidos vivos SUS apresentou o maior coeficiente de correlação para a RMM e a razões de condições potencialmente ameaçadores à vida, enquanto para a razão de *near miss* materno o maior coeficiente identificado foi o da variável início tardio do pré-natal. Observa-se que somente para as relações RMM e percentual de nascidos vivos no SUS, razão de condições potencialmente ameaçadores à vida e percentual de nascidos vivos no SUS e razão de condições potencialmente ameaçadores à vida e mãe solteira houve significância estatística.

**Tabela 1:** Índice de Moran bivariado para a correlação espacial entre a razão de mortalidade materna (RMM), razão de *near miss* materno e razão de condições potencialmente ameaçadores de vida e os indicadores demográficos, socioeconômicos, obstétricos e de acesso a pré-natal.

Indicadores *	RMM **		Razão de *near miss* materno ***		Razão de condições potencialmente ameaçadores de vida ***	
	%	Valor de p	%	Valor de p	%	Valor de p
Nascidos vivos atendidos no SUS	0,1492	0,049	-0,1087	0,106	0,1993	0,016
Nascidos vivos de mãe adolescente (< 20 anos)	0,0015	0,482	0,0335	0,426	-0,0511	0,229
Nascidos vivos de cor da pele preta ou parda	0,0534	0,286	-0,1426	0,054	0,0277	0,367
Nascidos vivos de mães com estado civil solteira	0,0078	0,489	-0,0549	0,253	0,1807	0,023
Nascidos vivos de mães com até 9 anos de estudo	-0,0874	0,136	-0,0213	0,358	0,0353	0,357
Nascidos vivos de primíparas	0,0292	0,327	-0,0505	0,318	-0,0151	0,458
Nascidos vivos por cesariana	0,0439	0,344	-0,0761	0,25	0,0766	0,177
Nascidos vivos de mães com início tardio do pré-natal ^#^	0,0296	0,340	0,1313	0,074	0,0514	0,272

* Indicadores calculados com dados do Sistema de Informação de Nascidos Vivos (SINASC);

** Indicador calculado com dados do Sistema de Informação de Mortalidade (SIM) e SINASC;

*** Indicadores calculados com dados do Sistema de Informações Hospitalares do Sistema Único de Saúde (SIH/SUS) e SINASC;

^#^ Início do pré-natal com idade gestacional > 12 semanas.

## Discussão

A RMM em usuárias do SUS no Município do Rio de Janeiro apresentou valor elevado e superior ao de 75/100 mil nascidos vivos, estabelecido como meta global nos Objetivos de Desenvolvimento Sustentável (ODS) ^5^, sendo muito maior do que a meta definida para o Brasil, de 30/100 mil nascidos vivos ^16^. A razão de *near miss* materno por 1.000 nascidos vivos também exibiu valor elevado, quase três vezes maior que o relatado em estudos produzidos com dados obtidos em prontuário hospitalar e que utilizaram a definição de caso de *near miss* materno da OMS ^17^^,^^18^. Para a razão de condições potencialmente ameaçadores à vida por 1.000 nascidos vivos, não foram encontrados estudos que tenham utilizado a definição de caso da OMS para analisar dados do SIH/SUS. Os resultados encontrados - relativos à frequência de casos de condições potencialmente ameaçadores à vida e de *near miss* materno, CID e procedimentos relacionados, e relação com a RMM - sugerem que a definição de caso de condições potencialmente ameaçadores à vida seja mais adequada para estimar a morbidade materna no SIH/SUS do que a definição de *near miss* materno.

Apesar da elevada RMM observada no Município do Rio de Janeiro, algumas RA com elevada vulnerabilidade social não apresentaram registros de óbitos maternos, mesmo utilizando um período de observação de três anos. Além disso, algumas áreas com menor população demonstraram flutuação nas suas taxas. Esses achados reforçam a importância da análise da morbidade materna de forma complementar ao estudo da mortalidade materna.

Pesquisas que avaliaram casos de *near miss* materno, como as realizadas pela Rede Brasileira de Vigilância da Morbidade Materna Grave ^19^ e pelo Departamento de Obstetrícia e Ginecologia do Zanzibar (Tanzânia) ^20^, demonstraram que o uso da definição de caso baseada em disfunção orgânica preconizada pela OMS é factível mediante a análise de prontuários. No entanto, a operacionalização por meio de sistemas de informação existentes pode ser difícil, devido à ausência de resultados de exames laboratoriais e à insuficiência de dados de monitoramento clínico, todos necessários para identificação dos casos ^21^. Entretanto, dada a importância do estudo da morbidade materna ^22^, esforços têm sido feitos para produzir estimativas de *near miss* materno em bases de dados administrativos, incluindo o SIH/SUS. Neste artigo, utilizaram-se os critérios adotados anteriormente por Nakamura-Pereira et al. ^7^ para identificação de casos de *near miss* materno em estudo realizado em um hospital universitário localizado no Município do Rio de Janeiro. De forma inovadora, foram identificados casos de condições potencialmente ameaçadores à vida adotando os critérios recomendados pela OMS. As condições potencialmente ameaçadores à vida fazem parte do espectro de gravidade da morbimortalidade materna e se baseiam em diagnósticos clínicos e procedimentos, sendo de mais fácil operacionalização no SIH/SUS.

A razão de *near miss* materno estimada para o Município do Rio de Janeiro foi muito superior às estimativas obtidas em estudos que utilizaram dados de prontuário para identificação de casos, próximas a 10/1.000 nascidos vivos ^17^^,^^18^. Valores entre 20 e 30/1.000 nascidos vivos foram observados em estudos que utilizaram definições pragmáticas, ou seja, baseadas em relatos maternos de condições marcadoras de gravidade (por exemplo, internação em UTI, transfusão sanguínea, histerectomia, eclâmpsia) ^23^^,^^24^, enquanto análises que utilizaram dados de AIH encontraram valores mais elevados, que variaram conforme a definição de caso utilizada: 52,9 internações para cada 1.000 partos em estudo no Paraná com utilização de três definições distintas ^25^; e 37,8/1.000 mulheres, em pesquisa realizada em Juiz de Fora (Minas Gerais), utilizando a definição de *near miss* materno da OMS ^26^.

Estudos utilizando o critério de condições potencialmente ameaçadores à vida da OMS não foram encontrados, o que dificulta a interpretação dos achados deste artigo. A razão de condições potencialmente ameaçadores à vida estimada para o Município do Rio de Janeiro, de 34,31/1.000 nascidos vivos, foi semelhante à relatada por Silva et al. ^25^, ao considerar o total de casos de morbidade materna identificados pelos critérios de Waterstone et al. ^27^ e Souza et al. ^19^ (39,1 por 1.000 nascidos vivos) e por Rosendo & Roncalli ^28^, utilizando critérios de Waterstone et al. ^27^ (36,67/1.000 mulheres).

Na comparação entre os casos de condições potencialmente ameaçadores à vida e *near miss* materno, três aspectos merecem destaque. Os casos de condições potencialmente ameaçadores à vida foram mais frequentes do que os de *near miss* materno, o que seria esperado, já que, segundo o gradiente da morbimortalidade materna, casos de *near miss* materno correspondem a uma pequena fração dos condições potencialmente ameaçadores à vida. Entretanto, a diferença no número de casos encontrados foi menor do que seria esperado, provavelmente porque alguns critérios utilizados para a identificação de casos de *near miss* materno não correspondem de fato aos critérios de disfunção orgânica preconizados pela OMS.

Na comparação dos diagnósticos e procedimentos identificados, verificamos um perfil dos casos de condições potencialmente ameaçadores à vida mais coerente com o perfil de mortalidade materna do Município do Rio de Janeiro e do país. Nos casos de *near miss* materno, os principais diagnósticos foram infecções, transtornos hemorrágicos e abortos, com apenas 7,48% dos diagnósticos relacionados a transtornos hipertensivos. A hipertensão é a principal causa de óbito materno no país, bem como a principal causa de *near miss* materno em outros estudos que avaliaram *near miss* materno no SIH ^25^ ou em pesquisas com definição pragmática ^23^^,^^24^. Quanto aos procedimentos, os mais frequentes na identificação dos casos de *near miss* materno foram “tratamento de complicações relacionadas predominantemente ao puerpério”, procedimento bastante inespecífico, seguido de “histerorrafia”, que não é uma boa *proxy* do critério de manejo clínico da OMS “histerectomia pós-parto”. Já na identificação dos casos de condições potencialmente ameaçadores à vida, os diagnósticos mais frequentes foram transtornos hipertensivos e distúrbios hemorrágicos, também relatados por Andrade et al. ^29^, enquanto os procedimentos mais comuns foram “tratamento de edema, proteinúria e transtornos hipertensivos na gravidez, parto e puerpério”, “tratamento cirúrgico da gravidez ectópica” e “laparotomia exploratória”.

Na análise espacial, a correlação da razão de condições potencialmente ameaçadores à vida com os indicadores demográficos, sociais e de acesso ao pré-natal foi mais próxima à observada para a RMM. Cabe destacar a correlação com o indicador “percentual de nascidos vivos no SUS”, que é entendido como um marcador de vulnerabilidade social, já que no Brasil o maior acesso a planos de saúde é observado em pessoas de maior escolaridade e renda ^30^^,^^31^. Esse foi o único indicador que apresentou correlação estatisticamente significativa com a RMM e razão de condições potencialmente ameaçadores à vida, enquanto a razão de *near miss* materno exibiu correlação negativa com diversos indicadores de vulnerabilidade, o que não era esperado.

Esses resultados sugerem que o condições potencialmente ameaçadores à vida é um indicador de morbidade materna mais adequado quando da utilização do SIH/SUS. Segundo recomendações da OMS, os indicadores de morbidade materna devem ser utilizados para o planejamento dos serviços de assistência obstétrica, identificando as áreas com maior necessidade de cuidados obstétricos e estimando os recursos e investimentos necessários para os cuidados à mulher no ciclo gravídico-puerperal ^1^^,^^2^.

Os indicadores de morbidade podem ser utilizados também para a análise do desempenho dos serviços de saúde ^32^, ao comparar os casos de morbidade e mortalidade, em que serviços que apresentam maior relação entre o número de casos de morbidade e mortalidade, bem como aqueles com menor letalidade dos casos graves, apresentam melhor desempenho ^1^^,^^2^^,^^6^.

Este estudo apresenta algumas limitações. A baixa cobertura de partos no SIH/SUS pode afetar diretamente as estimativas de *near miss* materno e condições potencialmente ameaçadores à vida, já que casos não registrados no SIH/SUS não são identificados, resultando em menores razões de *near miss* materno e condições potencialmente ameaçadores à vida nas áreas de menor cobertura. Dados de cobertura do SIH/SUS no Município do Rio de Janeiro não estão disponíveis. Para uma avaliação aproximada da cobertura do SIH/SUS na atenção obstétrica, calculou-se a proporção de nascidos vivos e óbitos fetais em internações obstétricas em maternidades do SUS, em comparação aos dados do SINASC e SIM, sistemas que apresentam cobertura elevada na cidade. Essa proporção, por RA, variou de 0,63% a 123,7% para nascidos vivos e de 0% a 197,6% para óbitos fetais, sendo as menores taxas observadas no Complexo do Alemão e no Complexo da Maré, ambas comunidades de baixa renda. É possível que erros no preenchimento do local de moradia expliquem a baixa cobertura observada nessas localidades, fazendo com que outras tenham cobertura superior a 100%. Outra explicação possível seria a maior ou menor dependência do orçamento dos diferentes hospitais em relação ao faturamento de AIH, resultando em menor preenchimento no sistema.

A segunda limitação refere-se à qualidade do preenchimento do SIH/SUS. Para identificar casos de *near miss* materno, a principal dificuldade no uso do SIH/SUS é a inexistência de informações sobre disfunção orgânica, sendo necessária a utilização de informações aproximadas, que podem resultar em erros de classificação e baixa acurácia.

Para localizar casos de condições potencialmente ameaçadores à vida, foram encontradas duas dificuldades específicas. O critério “internação após o parto > 7 dias” não é possível de ser operacionalizado, já que a data do parto não está disponível no SIH/SUS. Utilizando o tempo total de internação como *proxy*, identificou-se que muitas mulheres com internações longas não apresentavam qualquer tipo de complicação. É provável que essas mulheres estivessem acompanhando seus bebês internados - prática comum nos serviços públicos brasileiros -, o que resultaria em uma superestimação de casos de condições potencialmente ameaçadores à vida. A base de serviços profissionais do SIH/SUS, não utilizada neste estudo, contém um procedimento relativo ao acompanhamento de crianças internadas, que deve ser utilizado para evitar esse erro (procedimento 0802010024). Outra dificuldade foi a não identificação do procedimento “diária de unidade de terapia intensiva adulto” em nenhum dos anos analisados na base reduzida do SIH/SUS, já que ele é registrado na base de serviços profissionais. Como alternativa, foi utilizado o tipo de enfermaria em que as mulheres estavam internadas, para identificar casos por esse critério. Outra variável possível de ser usada é o número total de dias de UTI durante a internação.

Para analisar a correlação dos indicadores demográficos, sociais e de acesso ao pré-natal, bem como os de morbimortalidade materna, a análise com a RMM seria a mais confiável, já que se espera baixo sub-registro de óbitos em decorrência da boa vigilância do óbito materno realizada no Município do Rio de Janeiro. Entretanto, mesmo utilizando um período de três anos, algumas RA não apresentaram óbitos maternos, enquanto RA pequenas exibiram RMM elevada apesar da ocorrência de apenas dois óbitos no período. Além disso, não estavam disponíveis todas as variáveis relevantes para o estudo da mortalidade materna, e o não ajuste para essas outras variáveis pode ter afetado a correlação das variáveis observadas. Apesar dessas ressalvas, pode-se verificar um padrão de menor vulnerabilidade nas RA da zona sul da cidade, região com melhores condições sociais e econômicas e que apresentou menor RMM. Por outro lado, a zona oeste do município, região com baixa condição socioeconômica, apresentou proporção elevada de partos no SUS, bem como maior RMM. Para analisar os indicadores de razão de *near miss* materno e razão de condições potencialmente ameaçadores à vida, a correlação espacial apresenta limitações adicionais, relacionadas aos já citados problemas na cobertura de partos e qualidade de preenchimento: regiões com menor cobertura de partos SUS no SIH podem apresentar menor valor de razão de *near miss* materno e de razão de condições potencialmente ameaçadores à vida por menor identificação de casos; e a identificação de casos de *near miss* materno e condições potencialmente ameaçadores à vida pode ter falhas por má qualidade de preenchimento de CID e procedimentos.

Por fim, o período analisado não engloba os anos mais recentes disponíveis nos sistemas de informação utilizados. Entretanto, não houve modificação no padrão de mortalidade materna no Município do Rio de Janeiro no período de 2017 a 2019. Além disso, um dos objetivos do estudo era avaliar o uso de indicadores de morbidade materna de forma complementar à RMM, e não seria esperada uma mudança do perfil de morbidade em período tão curto.

## Conclusões

O Município do Rio de Janeiro apresenta indicadores de morbimortalidade materna elevados, com distribuição heterogênea na cidade, associada a marcadores de vulnerabilidade social. Considerando a elevada cobertura de assistência pré-natal e ao parto hospitalar existente na cidade, esses resultados sugerem problemas na qualidade dos serviços, bem como incapacidade da atenção obstétrica em atenuar desigualdades na determinação social da saúde materna.

A utilização do SIH/SUS para análise da morbidade materna, por meio da identificação de casos de *near miss* maetrno e condições potencialmente ameaçadores à vida, pode permitir uma análise mais ampliada da assistência à saúde da mulher, principalmente para as regiões que não apresentaram óbito materno no período analisado.

Casos de condições potencialmente ameaçadoras à vida, utilizados pela primeira vez neste estudo, apresentaram resultados mais condizentes com o perfil de mortalidade materna, bem como com pesquisas que identificaram casos de morbidade materna utilizando definições programáticas ou anteriores à definição recomendada atualmente pela OMS. Entretanto, novos estudos, principalmente comparando o SIH com dados de prontuários hospitalares, são necessários, tanto para estimar a cobertura global do SIH para internações obstétricas quanto para avaliar sua validade na identificação de casos de condições potencialmente ameaçadores à vida e *near miss* materno.
